# Supplementary material for: Efficacy and Sustainability of Diabetes-Specific Meal Replacement on Obese and Overweight Type-2 Diabetes Mellitus Patients: Study Approaches for a Randomised Controlled Trial and Impact of COVID-19 on Trial Progress
Source: Int J Environ Res Public Health. 2022 Apr 1;19(7):4188. doi: 10.3390/ijerph19074188 (PMC8998339; doi:10.3390/ijerph19074188)
Supplement: Supplementary file 1 [file ijerph-19-04188-s001.zip › Online Supplementary File S1 Information Sheet and Informed Consent Form.pdf]

INFORMATION SHEET FOR SUBJECT

Research Title:

Efficacy and sustainability of diabetes specific formula (Metabolic Sauver) on weight reduction and glycemic control among overweight and obese patients with Type 2 Diabetes Mellitus.

Introduction:

Type-2 diabetes mellitus (T2DM) has become increasingly epidemic globally. One of its main risks factors is obesity. Lifestyle intervention is a crucial aspect in managing obese or overweight T2DM patients. Meal replacement is proven to be an effective tool in achieving weight reduction and glycemic control in T2DM patients.

Purpose of Study:

Metabolic Sauver is meal replacement formulation designed specifically for diabetes patients. This research aims to study the efficacy and sustainability of Metabolic Sauver on weight reduction and glycemic control in obese or overweight T2DM patients. The effect of Metabolic Sauver on liver, renal and liver profile and lipid profile, satiety level, hormone changes, quality of life and cost effectiveness will also be monitored.

What will the study involve?

Participants aged 20-65 years will be interviewed on sociodemographic questions, medical history, lifestyle factors, physical activity questionnaire and food frequency questionnaire. Anthropometric measurements will be conducted to obtain baseline data for weight, height, BMI, waist circumference, hip circumference and body composition. 15.5 ml of blood will also be collected by a qualified phlebotomist to test on baseline HbA1c, fasting blood glucose, hormonal changes (insulin, adiponectin, leptin, ghrelin, obestatin and peptide YY), lipid, renal and liver profile. Satiety level will be monitored through visual analogue scale. Participants will also be assessed for quality of life with instruments like Audited Diabetes-dependent Quality of Life (ADDQOL), Diabetes Distress Scale (DDS) and Diabetes Treatment Satisfaction Questionnaire (DTSQ). Cost effectiveness will be assessed via EQ-5D-5L questionnaire.

Recruited participants will be randomized into two groups. Both groups will receive dietary consultation. On top of that, the intervention group will receive Metabolic Sauver to replace any one meal per day for 5 days a week. Both groups are required to keep a 3-day diet record throughout the study. A daily checklist on consumption of Metabolic Sauver will also be recorded for compliance monitoring. Participants will be required to return to the clinic on the 6th,12th, 24th, 36th and 48th week for follow up. Anthropometric measurements will be collected every time during follow up. 15.5 ml of blood samples will be collected for one more time at the 12th week and 48th week for post-intervention measurements.

Risks and Benefits:

Participants will be able to know their health status via blood sample testing and anthropometric measurements. Participants will also receive dietary consultation and/or meal replacement which help them to better manage their daily diet and might be able to reduce their weight and improve their glycemic control.

No direct risks will be involved in standard procedures of anthropometric measurements and blood collection. Metabolic Sauver meal replacement formulation does not have any reported side effects. Should there be any adverse or side effects, you can contact us and you will be arranged to Klinik Primer for further treatment immediately.

Do I have to take part?

Participation in this study is voluntary. If you agree to take part, then you will be asked to sign the "Informed Consent Form". You will be given a copy of the form and this Information Sheet. If you prefer not to take part, you do not have to give any reason.

Should you decide to participate, you can still withdraw from the study without any penalty at any time. Your data will not be used and will be discarded. The researcher may also remove you from the study for a variety of reasons. In this event, you will not be penalised.

Data & Confidentiality:

The data obtained from this study will be made into a publishable report in a collective manner with no reference to a specific individual. Access to the data is only by the research team and the REC UKM. Hence, your identity and data will be kept confidential and you are entitled to know about your data and result.

Payment and compensation:

You do not have to pay to participate in this study. A travel compensation of RM20 each will be given to you on the 1st and last visit.

Who can I ask about the study?

If you have any questions, you can direct them to the research team (details as listed below). You can also contact the REC UKM for clarifications.

Dr. Arimi Fitri mat Ludin
Biomedical Sciences Programme
Faculty of Health Sciences,
Universiti Kebangsaan Malaysia,
Jalan Raja Muda Abdul Aziz,
50300 Kuala Lumpur
Email: *arimifitri@ukm.edu.my*
Phone Number: 03-9289 8043

Lew Leong Chen
Biomedical Sciences Programme
Faculty of Health Sciences,
Universiti Kebangsaan Malaysia,
Jalan Raja Muda Abdul Aziz,
50300 Kuala Lumpur
Email: *lew0626@gmail.com*
Phone Number: 016-4109165

INFORMED CONSENT FORM

Research Title:Efficacy and Sustainabilityof diabetes specific formula (Metabolic Sauver) in weight reduction and glycemic control among overweight and obese patients with Type 2 Diabetes Mellitus

Participant's Name: ,

I, ……………………………………, IC No : …………………......

- have read the information in the Patient Information Sheet **including information regarding the risk in this study**
- have been given time to think about it and all of my questions have been answered to my satisfaction.
- understand that I may freely choose to withdraw from this study at anytime without reason and without repercussion
- understand that my anonymity will be ensured in the write-up.

I voluntarily agree to be part of this research study, to follow the study procedures, and to provide necessary information to the doctor, nurses, or other staff members, as requested.

………………………………. ……………………..

(Signature) (Date)

……………………………….<br>Witness (if any)	……………………………….<br>Researcher
……………………………….<br>(Signature)	……………………………….<br>(Signature)
……………………………….<br>(IC Number)	……………………………….<br>(IC Number)
…………………………..…<br>(Date)	……………………………….<br>(Date)
