# Supplementary material for: Efficacy and Sustainability of Diabetes-Specific Meal Replacement on Obese and Overweight Type-2 Diabetes Mellitus Patients: Study Approaches for a Randomised Controlled Trial and Impact of COVID-19 on Trial Progress
Source: Int J Environ Res Public Health. 2022 Apr 1;19(7):4188. doi: 10.3390/ijerph19074188 (PMC8998339; doi:10.3390/ijerph19074188)
Supplement: Supplementary file 1 [file ijerph-19-04188-s001.zip › Online Supplementary file S2- Metabolic Sauver Nutrition Sheet and Nutrition Content.pdf]

Supplementary File S2: *Metabolic Sauver* Nutrition Sheet and Nutrition Content.

Table S1. *Metabolic Sauver* Nutrition Facts

	**Per 100g**		**Per serving 75g**	
**Energy**	436	kcal	327	kcal
**Protein**	21.5	g	16.2	g
**Fat**	19.2	g	14.4	g
**Saturated fat**	13.8	g	10.35	g
**Trans fat**	0	g	0	g
**MCT**	9.6	g	7.2	g
**Carbohydrate**	49.5	g	37.2	g
**Dietary fiber**	10.5	g	7.95	g
**Isomaltulose**	7	g	5.25	g
**Lactose**	0	g	0	g
**Inositol**	200	mg	150	mg
**Taurine**	200	mg	150	mg
**L-Carnitine**	200	mg	150	mg
**Vitamin A**	1333	iu	1000.5	iu
**Vitamin D3**	200	iu	150	iu
**Vitamin E**	36	iu	27	iu
**Vitamin K1**	80	mcg	60	mcg
**Vitamin B1**	0.9	mg	0.675	mg
**Vitamin B2**	1.1	mg	0.825	mg
**Niacin**	13	mg	9.75	mg
**Vitamin B6**	2	mg	1.5	mg
**Vitamin B12**	3.9	mcg	2.925	mcg
**Vitamin C**	100	mg	75	mg
**Biotin**	500	mcg	375	mcg
**Folic acid**	250	mcg	187.5	mcg
**Choline**	200	mg	150	mg
**Pantothenic acid**	6	mg	4.5	mg
**Sodium**	406	mg	304.5	mg
**Potassium**	565	mg	424.5	mg
**Chloride**	528	mg	396	mg
**Calcium**	400	mg	300	mg
**Phosphorus**	400	mg	300	mg
**Magnesium**	200	mg	150	mg
**Iron**	5.4	mg	4.05	mg
**Zinc**	10	mg	7.5	mg
**Manganese**	2000	mcg	1500	mcg
**Copper**	1000	mcg	750	mcg
**Iodine**	200	mcg	150	mcg
**Selenium**	100	mcg	75	mcg
**Chromium**	100	mcg	75	mcg
**Molybdenum**	50	mcg	37.5	mcg
**Coenzyme Q10**	30	mg	22.5	mg
**Grape seed extract**	10	mg	7.5	mg
**Curcuma longa extract**	10	mg	7.5	mg
**Phytosterol**	10	mg	7.5	mg
**Gymnema extract**	10	mg	7.5	mg
**Fenugreek extract**	10	mg	7.5	mg
**Bitter melon extract**	10	mg	7.5	mg
**Guava leaf extract**	10	mg	7.5	mg
**Mulberry leaf extract**	10	mg	7.5	mg
**Cinnulin MS**	500	mg	375	mg

Table S2. Nutrition content in Metabolic Sauver

Nutrient	Benefits
Dietary fibre	Each serving= 5.3g fibre. High dietary fibre increases satiety level and reduces food intake.[1] It can also help improving glycemic control and lower risk of cardiovascular mortality in people with diabetes.[2]
Medium chain triglyceride	Reduced food intake by increasing energy expenditure leading to decreasing in body weight. Help increases insulin sensitivity by improving beta-cell function and reduce insulin resistance.[3]
Isomaltulose	A low glycemic index carbohydrate which is often used as sweeteners in food to replace sucrose. Shown effect of lowering postprandial glucose levels 20% - 52% compared to sucrose.[4]<br><br>Lower triglyceride levels in T2DM patients compared to sucrose.[5]
Vitamins and minerals	Contains a wide spectrum of vitamins and minerals for better nutritional values and to ensure patient receive adequate vitamins and minerals daily.
Isolated soy protein	Reduce serum LDL cholesterol and apolipoprotein B : apolipoprotein A-I ratio, hence reducing cardiovascular disease risks in T2DM patients.[6]<br><br>Able to improve glycemic control by reducing HbA1c and glucose level.[7]
Canola oil powder	Able to decrease serum low-density-lipoprotein cholesterol.[8]<br><br>Improved glycemic control as part of low-glycemic load diet, reducing HbA1c by 0.47%.[9]
Cinnamon extract (Cinnulin MS)	Possess anti-hyperglycemic properties and able to reduce fasting blood glucose.[10]<br><br>Cinnamon increases insulin sensitivity and glucose transport. It also inhibit retinol binding protein-4, an adipokine that contributes to insulin resistance.[11]
Fenugreek extract	Systematic review pooling 281 cases consuming fenugreek supplement has found out that fenugreek is an effective lipid lowering medicinal plant. It reduced the TG and LDL and increases HDL levels in diabetic subjects more effectively.[12]<br><br>It has also found to be effective in improving biomarkers of inflammation and oxidative stress as well as fasting insulin level of T2DM patients.[13, 14]

Gymnema extract	Gymnema sylvestre is a perennial woody vine extract that has long studied and used for its effects on improving glycemic control.[15]<br><br>Gymnema has been widely studied for its therapeutic potential in T2DM treatments due to its good hypoglycemic properties.[16, 17]
Bitter melon extract	Momordica charantia or bitter melon is commonly used as a traditional medicine in treating diabetes. Studies has found that bitter meon extract can stimulate insulin secretion from the endocrine pancreas, elicit glucose uptake in the liver and exert insulin-like effects on skeletal muscle cells.[18]<br><br>A study by Kim et. al. has shown that treatment with bitter melon extract for 12 weeks has reduced average fasting glucose level of the bitter melon group.[19]
Guava leaves extract	Guava leaves has been traditionally used to treat various symptoms of diabetes. A study has found out that guava leaf extract is able to suppresses the oxidative stress and inhibits the state of inflammation in diabetic mice and the results are almost similar to that of standard drug group using glibenclamide.[20]
Cucurma longa extract	Preclinical studies have proven that curcuminoids, the natural polyphenols extracted from the rhizome of turmeric, have antioxidant, anti-inflammatory and antitumor effects.[21-23]<br><br>Curcoma longa extracts has high antioxidant activity and were much more efficient in counteracting lipid peroxidation, particularly under pathological conditions and exerted greater protective effects against oxidative stress and inflammation.[22]<br><br>Cucurma Longa is suggested to be effective in both the prevention of diabetes mellitus and in attenuating the development of complications associated with the disease.[22, 24]
Mulberry leaves extract	Leaves from mulberry or *Morus alba* is widely used in Asian country for its antidiabetic due to its properties attributed to the presence of 1-deoxynojirimycin (DNJ). It is also found to help improving the lipid profile and reduce the cholesterol levels.[25, 26]
Grape seed extract	Grape seed extract is commonly studied for its anticancer and chemopreventive properties.[27] It also have a broad spectrum against oxidative stress which brings a series of potential health benefits include protection against oxidative damage, and anti-diabetic, anti-cholesterol, and anti-platelet functions.[28]

REFERENCES

1. Yu, K.; Ke, M.-Y.; Li, W.-H.; Zhang, S.-Q.; Fang, X.-C., The impact of soluble dietary fibre on gastric emptying, postprandial blood glucose and insulin in patients with type 2 diabetes. *Asia Pacific journal of clinical nutrition* **2014**.
2. Burger, K. N. J.; Beulens, J. W. J.; van der Schouw, Y. T.; Sluijs, I.; Spijkerman, A. M. W.; Sluik, D.; Boeing, H.; Kaaks, R.; Teucher, B.; Dethlefsen, C.; Overvad, K.; Tjønneland, A.; Kyrø, C.; Barricarte, A.; Bendinelli, B.; Krogh, V.; Tumino, R.; Sacerdote, C.; Mattiello, A.; Nilsson, P. M.; Orho-Melander, M.; Rolandsson, O.; Huerta, J. M.; Crowe, F.; Allen, N.; Nöthlings, U., Dietary fiber, carbohydrate quality and quantity, and mortality risk of individuals with diabetes mellitus. *PloS one* **2012,** *7* (8), e43127-e43127.
3. Han, J. R.; Deng, B.; Sun, J.; Chen, C. G.; Corkey, B. E.; Kirkland, J. L.; Ma, J.; Guo, W., Effects of dietary medium-chain triglyceride on weight loss and insulin sensitivity in a group of moderately overweight free-living type 2 diabetic Chinese subjects. *Metabolism* **2007,** *56* (7), 985-991.
4. Maresch, C. C.; Petry, S. F.; Theis, S.; Bosy-Westphal, A.; Linn, T., Low Glycemic Index Prototype Isomaltulose—Update of Clinical Trials. *Nutrients* **2017,** *9* (4), 381.
5. Brunner, S.; Holub, I.; Theis, S.; Gostner, A.; Melcher, R.; Wolf, P.; Amann-Gassner, U.; Scheppach, W.; Hauner, H., Metabolic Effects of Replacing Sucrose by Isomaltulose in Subjects With Type 2 Diabetes. *Diabetes Care* **2012,** *35* (6), 1249.
6. Pipe, E. A.; Gobert, C. P.; Capes, S. E.; Darlington, G. A.; Lampe, J. W.; Duncan, A. M., Soy protein reduces serum LDL cholesterol and the LDL cholesterol: HDL cholesterol and apolipoprotein B: apolipoprotein AI ratios in adults with type 2 diabetes. *The Journal of nutrition* **2009,** *139* (9), 1700-1706.
7. Li, Z.; Hong, K.; Saltsman, P.; DeShields, S.; Bellman, M.; Thames, G.; Liu, Y.; Wang, H.; Elashoff, R.; Heber, D., Long-term efficacy of soy-based meal replacements vs an individualized diet plan in obese type II DM patients: relative effects on weight loss, metabolic parameters, and C-reactive protein. *European Journal of Clinical Nutrition* **2005,** *59* (3), 411.
8. Bierenbaum, M. L.; Reichstein, R. P.; Watkins, T. R.; Maginnis, W. P.; Geller, M., Effects of canola oil on serum lipids in humans. *Journal of the American College of Nutrition* **1991,** *10* (3), 228-233.
9. Jenkins, D. J. A.; Kendall, C. W. C.; Vuksan, V.; Faulkner, D.; Augustin, L. S. A.; Mitchell, S.; Ireland, C.; Srichaikul, K.; Mirrahimi, A.; Chiavaroli, L.; Blanco Mejia, S.; Nishi, S.; Sahye-Pudaruth, S.; Patel, D.; Bashyam, B.; Vidgen, E.; de Souza, R. J.; Sievenpiper, J. L.; Coveney, J.; Josse, R. G.; Leiter, L. A., Effect of Lowering the Glycemic Load With Canola Oil on Glycemic Control and Cardiovascular Risk Factors: A Randomized Controlled Trial. *Diabetes Care* **2014,** *37* (7), 1806.
10. Kirkham, S.; Akilen, R.; Sharma, S.; Tsiami, A., The potential of cinnamon to reduce blood glucose levels in patients with type 2 diabetes and insulin resistance. *Diabetes, Obesity and Metabolism* **2009,** *11* (12), 1100-1113.
11. Qin, B.; Panickar, K. S.; Anderson, R. A., Cinnamon: Potential Role in the Prevention of Insulin Resistance, Metabolic Syndrome, and Type 2 Diabetes. *Journal of Diabetes Science and Technology* **2010,** *4* (3), 685-693.
12. Heshmat-Ghahdarijani, K.; Mashayekhiasl, N.; Amerizadeh, A.; Teimouri Jervekani, Z.; Sadeghi, M., Effect of fenugreek consumption on serum lipid profile: A systematic review and meta-analysis. *Phytotherapy Research* **2020,** *34* (9), 2230-2245.
13. Tavakoly, R.; Maracy, M. R.; Karimifar, M.; Entezari, M. H., Does fenugreek (Trigonella foenum-graecum) seed improve inflammation, and oxidative stress in patients with type 2 diabetes mellitus? A parallel group randomized clinical trial. *European Journal of Integrative Medicine* **2018,** *18*, 13-17.

14. Najdi, R. A.; Hagras, M. M.; Kamel, F. O.; Magadmi, R. M. J. A. h. s., A randomized controlled clinical trial evaluating the effect of Trigonella foenum-graecum (fenugreek) versus glibenclamide in patients with diabetes. **2019,** *19* (1), 1594-1601.
15. Shanmugasundaram, E.; Rajeswari, G.; Baskaran, K.; Kumar, B. R.; Shanmugasundaram, K. R.; Ahmath, B. K. J. J. o. e., Use of Gymnema sylvestre leaf extract in the control of blood glucose in insulin-dependent diabetes mellitus. **1990,** *30* (3), 281-294.
16. Khan, F.; Sarker, M. M. R.; Ming, L. C.; Mohamed, I. N.; Zhao, C.; Sheikh, B. Y.; Tsong, H. F.; Rashid, M. A., Comprehensive Review on Phytochemicals, Pharmacological and Clinical Potentials of Gymnema sylvestre. **2019,** *10* (1223).
17. Tiwari, P.; Ahmad, K.; Hassan Baig, M. J. C. p. d., Gymnema sylvestre for diabetes: From traditional herb to future's therapeutic. **2017,** *23* (11), 1667-1676.
18. Joseph, B.; Jini, D., Antidiabetic effects of Momordica charantia (bitter melon) and its medicinal potency. *Asian Pacific Journal of Tropical Disease* **2013,** *3* (2), 93-102.
19. Kim, S. K.; Jung, J.; Jung, J. H.; Yoon, N.; Kang, S. S.; Roh, G. S.; Hahm, J. R., Hypoglycemic efficacy and safety of Momordica charantia (bitter melon) in patients with type 2 diabetes mellitus. *Complementary Therapies in Medicine* **2020,** *52*, 102524.
20. Jayachandran, M.; Vinayagam, R.; Ambati, R. R.; Xu, B.; Chung, S. S. M., Guava Leaf Extract Diminishes Hyperglycemia and Oxidative Stress, Prevents <i><i>β</i></i>-Cell Death, Inhibits Inflammation, and Regulates NF-kB Signaling Pathway in STZ Induced Diabetic Rats. *BioMed Research International* **2018,** *2018*, 4601649.
21. Vecchi Brumatti, L.; Marcuzzi, A.; Tricarico, P. M.; Zanin, V.; Girardelli, M.; Bianco, A. M., Curcumin and inflammatory bowel disease: potential and limits of innovative treatments. *Molecules (Basel, Switzerland)* **2014,** *19* (12), 21127-53.
22. Margină, D.; Olaru, O. T.; Ilie, M.; Grădinaru, D.; GuȚu, C.; Voicu, S.; Dinischiotu, A.; Spandidos, D. A.; Tsatsakis, A. M., Assessment of the potential health benefits of certain total extracts from Vitis vinifera, Aesculus hyppocastanum and Curcuma longa. *Exp Ther Med* **2015,** *10* (5), 1681-1688.
23. Ibrahim, J.; Kabiru, A. Y.; Abdulrasheed-Adeleke, T.; Lawal, B.; Adewuyi, A. H. J. J. o. T. U. f. S., Antioxidant and hepatoprotective potentials of curcuminoid isolates from turmeric (Curcuma longa) rhizome on CCl4-induced hepatic damage in Wistar rats. **2020,** *14* (1), 908-915.
24. Karłowicz-Bodalska, K.; Han, S.; Freier, J.; Smoleński, M.; Bodalska, A. J. A. P. P. D. R., Curcuma longa as medicinal herb in the treatment of diabetic complications. **2017,** *74* (2), 605-610.
25. Amin, A. R.; Kassab, R. B.; Abdel Moneim, A. E.; Amin, H. K., Comparison Among Garlic, Berberine, Resveratrol, Hibiscus sabdariffa, Genus Zizyphus, Hesperidin, Red Beetroot, Catha edulis, Portulaca oleracea, and Mulberry Leaves in the Treatment of Hypertension and Type 2 DM: A Comprehensive Review. *Natural Product Communications* **2020,** *15* (4), 1934578X20921623.
26. Sheng, Y.; Liu, J.; Zheng, S.; Liang, F.; Luo, Y.; Huang, K.; Xu, W.; He, X. J. F.; function, Mulberry leaves ameliorate obesity through enhancing brown adipose tissue activity and modulating gut microbiota. **2019,** *10* (8), 4771-4781.
27. Kaur, M.; Agarwal, C.; Agarwal, R., Anticancer and Cancer Chemopreventive Potential of Grape Seed Extract and Other Grape-Based Products. *The Journal of Nutrition* **2009,** *139* (9), 1806S-1812S.
28. Kwatra, B. J. W. J. P. R., A review on potential properties and therapeutic applications of grape seed extract. **2020,** *9*, 2519-2540.
